# Transcriptomic Analysis of High Fat Diet Fed Mouse Brain Cortex

**DOI:** 10.3389/fgene.2019.00083

**Published:** 2019-02-19

**Authors:** Gwangho Yoon, Kyung A Cho, Juhyun Song, Young-Kook Kim

**Affiliations:** ^1^Department of Anatomy, Chonnam National University Medical School, Jeollanam-do, South Korea; ^2^Department of Biochemistry, Chonnam National University Medical School, Jeollanam-do, South Korea; ^3^Department of Biomedical Sciences, Center for Creative Biomedical Scientists at Chonnam National University, Jeollanam-do, South Korea

**Keywords:** non-coding RNAs, long non-coding RNAs, circular RNAs, high fat diet, brain cortex

## Abstract

High fat diet can lead to metabolic diseases such as obesity and diabetes known to be chronic inflammatory diseases with high prevalence worldwide. Recent studies have reported cognitive dysfunction in obese patients is caused by a high fat diet. Accordingly, such dysfunction is called “type 3 diabetes” or “diabetic dementia.” Although dysregulation of protein-coding genes has been extensively studied, profiling of non-coding RNAs including long non-coding RNAs (lncRNAs) and circular RNAs (circRNAs) has not been reported yet. Therefore, the objective of this study was to obtain profiles of diverse RNAs and determine their patterns of alteration in high fat fed brain cortex compared to normal brain cortex. To investigate regulatory roles of both coding and non-coding RNAs in high fat diet brain, we performed RNA sequencing of ribosomal RNA-depleted RNAs and identified genome-wide lncRNAs and circRNAs expression and co-expression patterns of mRNAs in high fat diet mouse brain cortex. Our results showed expression levels of mRNAs related to neurogenesis, synapse, and calcium signaling were highly changed in high fat diet fed cortex. In addition, numerous differentially expressed lncRNAs and circRNAs were identified. Our study provides valuable expression profiles and potential function of both coding and non-coding RNAs in high fat diet fed brain cortex.

## Introduction

It is known that metabolic diseases such as diabetes and obesity (Grundy, 2005) can trigger brain dysfunction and contribute to the development of dementia (Parrott and Greenwood, 2007; Craft, 2009). Epidemiologic studies have demonstrated the association between obesity caused by high fat diet intake and increased risk of Alzheimer’s disease (AD) accompanied by a cognitive decline (Luchsinger et al., 2002; Greenwood and Winocur, 2005). In addition, clinical studies have reported that diabetes patients have increased risk of AD compared to normal subjects (Brands et al., 2005) and 80% of AD patients have high fasting blood glucose level (Janson et al., 2004). Furthermore, a significant association between high fat diet and cognitive dysfunction has been reported (Winocur et al., 2005; Cordner and Tamashiro, 2015). Some epidemiological studies have shown that high fat diet intake of mostly omega-6 and saturated fatty acid can trigger impaired performance for a cognitive task (Morris et al., 2004; Zhang et al., 2006). On the other hand, a clinical study has reported that lower fat diet consisting of omega-3 fatty acids could suppress a cognitive decline in healthy older subjects (Uranga et al., 2010). In animal models, high fat diet feeding leads to a reduction of hippocampal dendrite integrity, activation of microglia (Granholm et al., 2008), brain insulin resistance, and cognitive impairment in mice (Kothari et al., 2017). It can also worsen learning ability and suppress long-term potentiation (LTP) associated with memory function in the rat (Stranahan et al., 2008). Several studies have highlighted that high fat diet feeding can inhibit synaptic plasticity in the prefrontal cortex (Val-Laillet et al., 2011) and cause reduction of BDNF protein, a critical protein for promoting LTP and enhancing synaptic plasticity in prefrontal cortex known to be the key brain area in learning and memory function (Kanoski et al., 2007; Noble et al., 2011). Given these previous studies, the study on high fat diet fed brain cortex to find detailed mechanisms and related genes in high fat-induced cognitive decline is important.

Although previous studies investigated the alteration of various protein-coding genes in high fat diet fed brain, they did not investigate non-coding RNAs including long non-coding RNAs (lncRNAs) and circular RNAs (circRNAs) (Lee et al., 2010; Nam et al., 2017). Non-coding RNAs are divided into lncRNAs (>200 nt) and small non-coding RNAs (20–200 nt) (Wapinski and Chang, 2011). It has been estimated that there are more than 50,000 lncRNA genes in the human genome (Managadze et al., 2013). lncRNAs are generally expressed in a tissue-specific manner. They are also detected in body fluids (Mercer et al., 2009). Nucleus-localized lncRNAs play regulatory roles as transcriptional co-regulators by binding to transcription factors in a complex (Ulitsky and Bartel, 2013). These lncRNAs control the expression of nearby genes on the same allele *in cis* or those genes at another genomic location *in trans* through transcription control (Elling et al., 2016) and chromatin remodeling (Wilusz et al., 2009). On the other hand, cytoplasmic lncRNAs primarily regulate mRNA expression by blocking the action of microRNA (miRNA) (Noh et al., 2018). Previous studies have reported that many lncRNAs are expressed in brain regions including cortex, cerebellum, and hippocampus (Kadakkuzha et al., 2015; Molyneaux et al., 2015). These lncRNAs regulate diverse pathological processes in neuronal diseases (Ng et al., 2013; Quan et al., 2017).

circRNAs were firstly discovered in RNA viruses (Sanger et al., 1976). They are also involved in the dynamic regulation of gene expression in diverse physiological processes. circRNAs can control the expression of parental genes (Li et al., 2015), regulate alternative splicing (Ashwal-Fluss et al., 2014), modulate RNA–protein interactions (Du et al., 2016), and act as miRNA sponges (Zheng et al., 2016). Importantly, plentiful circRNAs have been found in mammalian brains (You et al., 2015). They are associated with human neurodegenerative diseases (Kumar et al., 2017). Since most exonic circRNAs with half-lives of more than 48 hrs (Jeck and Sharpless, 2014) are much more stable than linear RNAs (Salzman, 2016), circRNAs have potential as molecular markers for disease diagnosis and treatment.

The objective of this study was to acquire expression map of diverse coding and non-coding RNAs including lncRNAs and circRNAs and infer their potential functions in high fat diet fed brain cortex. We performed RNA sequencing and analyzed their expression changes in high fat diet brain. Functional analyses identified diverse biological processes affected by high fat diet. Results of our analyses provide important information of coding and non-coding RNAs in high fat diet brain.

## Materials and Methods

### Sample Preparation

Male C57BL/6 mice (Orient) were obtained at 8 weeks of age. The mice were fed with either a conventional diet or a diet enriched with fat (60% wt/wt; Bio-Serv) for 8 weeks. At 16 weeks old, the high fat diet fed mice showed increased weight and impaired glucose tolerance. To obtain the brain cortexes, mice were sacrificed under ether anesthesia. The experiment was carried out in accordance with the recommendations of ‘96 Guidance for Animal Experiments,’ established by the ‘Animal Ethics Committee’ at Chonnam National University, and the protocol was approved by the ‘Animal Ethics Committee’ at Chonnam National University.

### RNA Sequencing

Total RNAs from brain cortexes were extracted using TRIzol reagent (Thermo Fisher) and a tissue homogenizer (Omni). The integrity of total RNA was checked using Agilent 2100 BioAnalyzer (Agilent). RNA integrity number (RIN) of all samples was greater than 9. RNA samples were treated with Ribo-Zero Gold rRNA Removal Kit (Illumina) and library for RNA sequencing was prepared using TruSeq Stranded Total RNA Kit (Illumina). The library was paired-end sequenced on HiSeq 2500 system (Illumina) with 100 sequencing cycles.

### Analysis of RNA Sequencing Data

The quality of sequence reads produced from the sequencer was checked by FastQC^1^ and sequences with low quality were trimmed using Trimmomatic (Bolger et al., 2014). To analyze expression levels of mRNAs and lncRNAs, we utilized two different pipelines. First, those trimmed sequences were aligned into mouse genome (mm10) using STAR aligner (Dobin et al., 2013). Normalized values of Fragments Per Kilobase of transcript per Million mapped reads (FPKM) were calculated using Cuffnorm (Trapnell et al., 2012) based on recent basic gene annotation in GENCODE (Release M17, GRCm38.p6) (Harrow et al., 2006). We excluded genes from further analyses if their average FPKM values were less than 1 or FPKM was 0 in any sample. The *t*-test was applied to select those genes with significant expression change between normal and high fat diet groups. Second, the same trimmed sequences were analyzed to quantify expression levels of transcripts using Salmon tool (Patro et al., 2017) and edgeR package (Robinson et al., 2010) was used to detect genes with significant expression changes. Results from these two approaches were combined, and only those genes with significant expression changes in both pipelines were selected for further analyses.

For unsupervised hierarchical clustering, Cluster 3.0 was used for clustering (de Hoon et al., 2004) and Java Treeview was used for visualization (Saldanha, 2004). We selected 6,889 mRNAs and lncRNAs with average FPKM values higher than 10. In Cluster 3.0, FPKM values were log-transformed. Genes and arrays were median-centered and normalized. We used complete linkage analysis for hierarchical clustering with the centered-correlation method.

### Functional Analysis of mRNAs

For the group of genes differentially expressed between cortexes from normal and high fat diet groups, we performed gene ontology (GO) enrichment analysis at Molecular Signatures Database (MSigDB) (Liberzon et al., 2011; The Gene Ontology Consortium, 2017). For each group of genes with increased or decreased expression, enriched pathways were analyzed in MSigDB using Kyoto Encyclopedia of Genes and Genomes (KEGG) pathway database (Kanehisa et al., 2016).

### Measurement of Protein Level by Western Blot

The brain cortexes of high fat diet fed mice were homogenized with ice-cold RIPA buffer (Translab). After quantification using BCA assay kit (Thermo Scientific), protein (30 μg) was separated on 6–15% SDS-polyacrylamide gel depending on the size of proteins. The gel was transferred onto methanol-activated polyvinylidene difluoride (PVDF) membrane. The membrane was blocked with 5% skim milk (BD Bioscience) or bovine serum albumin (Sigma-Aldrich) prepared in Tris-buffered saline-tween [TBS-T; 20 nM Tris (pH7.2), 150mM NaCl, 0.1% Tween 20] for 1 h followed by incubation with primary antibody for overnight at 4°C. After incubation with secondary antibody for 1 h, the blot was visualized using ECL solution (Thermo Scientific) and Fusion Solo (Vilber). The information of antibodies is included in the Supplementary Data.

### Measurement of RNA Level by Polymerase Chain Reaction (PCR)

Total RNA was reverse transcribed into complementary DNA (cDNA) using RevertAid reverse transcriptase (Thermo Scientific). PCR was performed with the cDNA using Phusion DNA polymerase (Thermo Scientific) in Mastercycler nexus X2 (Eppendorf). The PCR products were separated by gel electrophoresis and the bands were quantified using ImageJ (Schneider et al., 2012). The expression level of each lncRNA and circRNA was normalized to the level of Gapdh (Glyceraldehyde 3-phosphate dehydrogenase). The list of PCR primers is included in the Supplementary Data.

### Analysis of circRNAs Expression

We used DCC algorithm to detect reads containing back-spliced junctions from the sequencing data (Cheng et al., 2016). We only selected exonic circRNAs composed of only exonic sequences of genes. Calculated reads for each circRNA candidate were normalized by the number of total circRNA reads. Those circRNAs with average read numbers among samples greater than 2 were selected for further analyses. In the case of selecting differentially expressed circRNAs, only circRNAs with average read numbers greater than 10 were used.

### Confirmation of the Circular Structure of circRNAs

To confirm the circular structure of circRNAs, total RNA was treated with RNase R (Epicentre) which remove linear RNAs. The mixture was incubated at 37°C for 20 min and inactivated at 95°C for 3 min. The reactant was reverse transcribed into cDNA and PCR-amplified. The PCR products were sequenced to confirm the expected back-splice junctions of circRNAs. The list of PCR primers is included in the Supplementary Data.

## Results

### Transcriptome Analysis of Brain Cortexes of Mice Fed With High Fat Diet

**FIGURE 1:**
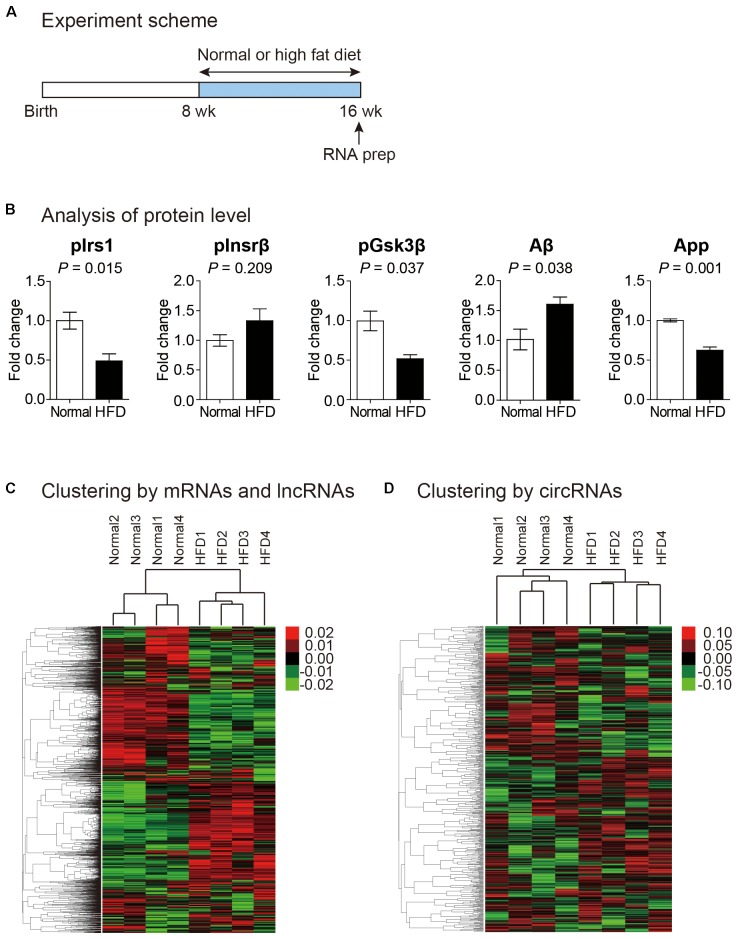
Transcriptomic analysis of the cortex of mouse fed with high fat diet. **(A)** Experiment scheme to analyze the transcriptome in normal and high fat diet fed mice. **(B)** Analysis of proteins related to insulin resistance and cognitive impairment. Phosphorylated form of insulin receptor substrate 1 (pIrs1) and that of insulin receptor beta (pInsrβ) were measured to confirm insulin resistance. Phosphorylated form of glycogen synthase kinase 3 beta (pGsk3β), amyloid beta (Aβ), and amyloid precursor protein (App) were measured to indicate cognitive impairment. The data in Supplementary Figure S2 were used for the quantitation. *P* value was calculated by two-tailed *t*-test (*n* = 2 in each group). **(C,D)** Heat maps of expression profile analyzed from unsupervised hierarchical clustering of mRNAs and lncRNAs **(C)** and circRNAs **(D)** were shown. Color bars were included to illustrate relative expression. In both maps, each sample group was clustered properly.

Previous studies have suggested that high fat diet affects gene expression profile of brain (Lee et al., 2010; Nam et al., 2017). However, those studies only measured protein-coding genes. No study has dealt with non-coding RNAs. To comprehensively analyze the effect of high fat diet on transcriptome profile of brain, we fed mice of 8-week-old with high fat diet for an additional 8 weeks (Figure 1A). The weight of these mice increased significantly compared to that of control mice with normal diet, and a problem in the maintenance of glucose homeostasis was confirmed with the glucose tolerance test (Supplementary Figure S1). Moreover, the phosphorylated form of insulin receptor substrate 1 (Irs1) decreased while that of insulin receptor beta (Insrβ) increased in the brain cortexes of mice fed with high fat diet confirming the insulin resistance in the brain of these mice (Figure 1B). In addition to the insulin resistance, we also confirmed the expression change of genes related to cognitive decline. We found the reduction of phosphorylated form of glycogen synthase kinase 3 beta (Gsk3β), the increase of amyloid beta (Aβ) peptide, and the decrease of amyloid precursor protein (App) in the brain cortex of mice fed with high fat diet (Figure 1B). The reduction in the inhibitory phosphorylation of Gsk3β aggravates cognitive impairment (Llorens-Martin et al., 2014). The Aβ peptide is processed from proteolytic cleavage of App, and the accumulation of Aβ in the brain is the most critical factor in the AD (O’Brien and Wong, 2011).

We prepared rRNA-depleted total RNAs for brain cortex samples (from 4 normal and 4 high fat diet fed mice) and performed RNA sequencing analysis. We removed sequencing reads with low quality and measured mRNA and lncRNA levels by STAR-Cuffnorm pipeline (see Materials and Methods) (Trapnell et al., 2012; Dobin et al., 2013). To analyze expression levels of circRNAs, we used DCC algorithm (see Materials and Methods) (Cheng et al., 2016).

To confirm that samples in the same experimental group showed similar expression pattern, we performed unsupervised hierarchical clustering analysis. When expression levels of mRNAs and lncRNAs were analyzed, samples from the same group (normal or high fat diet) clearly clustered together, suggesting the reliability of samples that we made (Figure 1C). The same pattern of clustering was also observed when the expression of circRNAs was analyzed, although correlation among samples from the same group was lower (Figure 1D). Therefore, we combined expression levels from the four samples in each group and performed subsequent analyses.

Quantitation of transcripts level using diverse bioinformatics algorithms can result in different results due to different performance and sensitivity of these algorithms (Everaert et al., 2017). To circumvent this issue, we used transcript quantitation based on an additional analysis pipeline, Salmon-edgeR (see Materials and Methods) (Robinson et al., 2010; Patro et al., 2017). We selected 286 mRNAs and 24 lncRNAs whose expression levels were changed significantly after high fat diet in both analysis pipeline, STAR-Cuffnorm and Salmon-edgeR. Using these mRNAs and lncRNAs sets, we performed the following analyses.

### Analysis of mRNAs Change in the Brain Cortex After High Fat Diet

**FIGURE 2:**
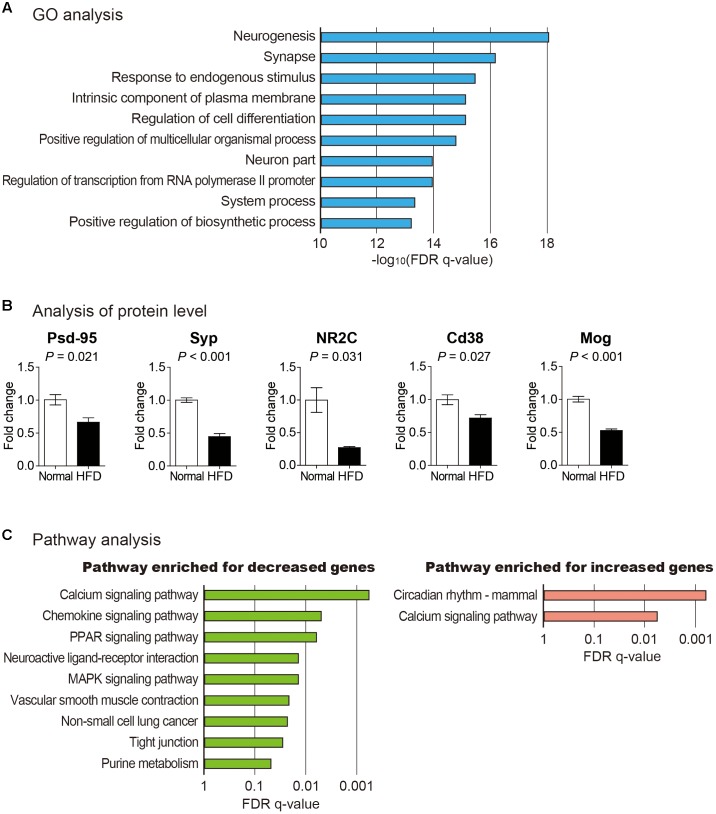
Analysis of protein-coding mRNAs in the brain cortex of mouse fed with high fat diet. **(A)** GO analysis for differentially expressed mRNAs between cortexes from normal and high fat diet fed mice. Top 10 significantly enriched GO terms based on their false discovery rate (FDR) *q*-value are shown. **(B)** Analysis of proteins related to synaptic plasticity, calcium signaling, and myelination. Postsynaptic density protein 95 (Psd-95), synaptophysin (Syp), and glutamate receptor NMDAR2C (NR2C) were measured to confirm reduced synaptic plasticity. Cd38 was measured to show the alternation in calcium homeostasis. And myelin oligodendrocyte glycoprotein (Mog) was measured as a marker of myelination. The data in Supplementary Figure S2 were used for the quantitation. *P*-value was calculated by two-tailed *t*-test (*n* = 2 in each group). **(C)** Analyses to identify enriched pathway among decreased and increased mRNAs after high fat diet feeding, respectively. Only those pathways with FDR *q*-value less than 0.05 are shown.

To infer the effect of high fat diet on the brain cortex, we analyzed functions of differentially expressed mRNAs (Supplementary Table S1). For this, GO enrichment analysis and pathway analysis based on KEGG database were performed (Figure 2) (Liberzon et al., 2011; Kanehisa et al., 2016; The Gene Ontology Consortium, 2017). GO analysis for coding genes (both increased and decreased) showed that neuron-related terms including neurogenesis and synapse were highly enriched in this gene group (Figure 2A and Supplementary Table S2). To confirm the result of GO analysis, we checked whether several marker genes related to synaptic function changed in their expression. The protein level of postsynaptic density protein 95 (Psd-95) and synaptophysin (Syp) were reduced in the brain cortex from mice fed with high fat diet (Figure 2B). Psd-95, a synaptic plasticity-related protein, is highly enriched at the postsynaptic site of excitatory synapse and organizes various signaling at the postsynapse (Kim and Sheng, 2004). Syp was shown to regulate activity-dependent synapse formation and is required for efficient endocytosis of synaptic vesicles in cultured hippocampal neurons (Tarsa and Goda, 2002; Kwon and Chapman, 2011). Hence, the reduction of Psd-95 and Syp suggests a synapse dysfunction in the brain cortex from the mice with high fat diet, which confirms the result of GO analysis and the cognitive decline in this model.

When pathway analysis based on KEGG database was performed for decreased mRNAs in the cortex of high fat diet fed brain, calcium signaling pathway was identified to be the most highly enriched (Figure 2C). Interestingly, a similar analysis for increased mRNAs also identified calcium signaling pathway as the most significantly changed one. By manually inspecting each gene from these groups, we found that expression levels of mRNAs up-regulating calcium signaling were decreased while those of mRNAs down-regulating calcium signaling were increased, thus diminishing this signaling pathway. One of these significantly decreased genes, Cd38, is a membrane-bound glycoprotein associated with the activity of ADP-ribosyl cyclase (Khoo et al., 2000) (Supplementary Table S2). Cd38/cyclic ADP-ribose signaling is critical in the regulation of calcium homeostasis in cells (Deshpande et al., 2005). We confirmed decreased expression of Cd38 in the brain cortex from the mice fed with high fat diet (Figure 2B). Based on our results, decreased expression of Cd38 and other genes in high fat diet fed mouse brain cortex might be involved in impaired calcium signaling in brain cortex.

### Analysis of lncRNAs Change in the Brain Cortex After High Fat Diet

**FIGURE 3:**
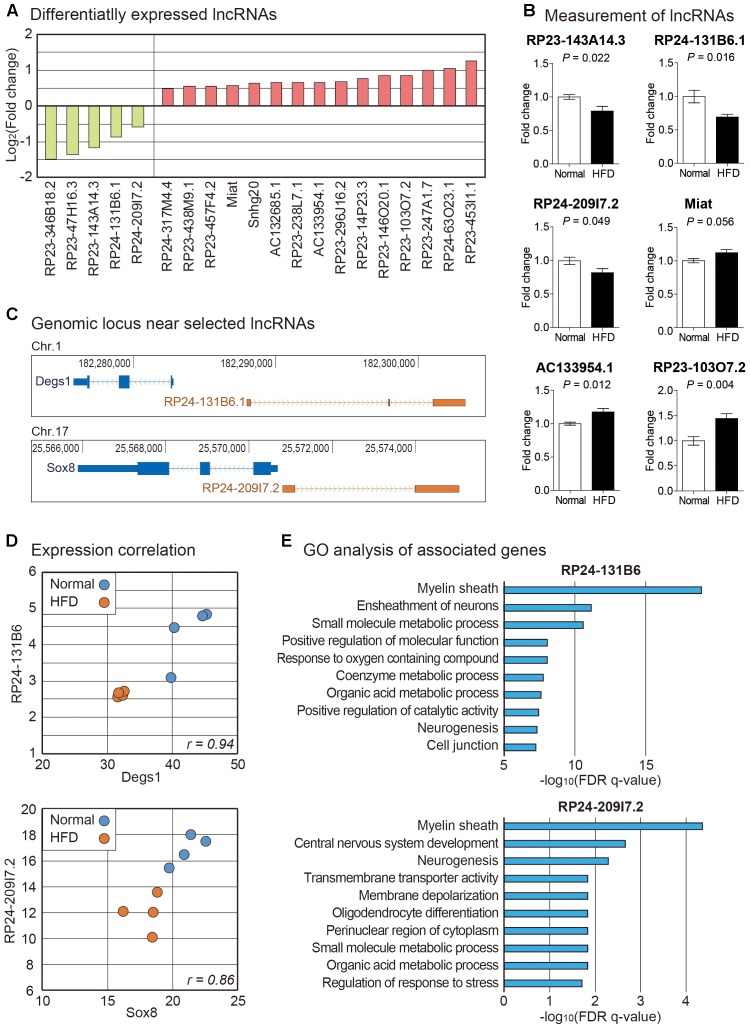
Analysis of lncRNAs in the brain cortex of mouse fed with high fat diet. **(A)** List of lncRNAs significantly changed in high fat diet fed mouse cortex. We only selected those lncRNAs significantly changed in both STAR-Cuffnorm and Salmon-edgeR pipelines (see Materials and Methods). **(B)** Measurement of lncRNAs by PCR. Six lncRNAs were randomly selected among those in **(A)** to confirm their expression change. The data in Supplementary Figure S3 were used for the quantitation. *P*-value was calculated by two-tailed *t*-test (*n* = 4 in each group). **(C)** Genomic information of lncRNAs, RP24-131B6.1 and RP24-209I7.2, and their nearby protein-coding genes were obtained from UCSC Genome Browser (https://genome.ucsc.edu). Thick boxes indicate sequences of open reading frame while narrow boxes indicate those of untranslated region. Solid lines indicate intronic sequences where arrows show the direction of transcription. **(D)** The expression correlation was indicated with scatter plot based on FPKM values of mRNA-lncRNA pairs in **(C)**. Each dot indicates the expression from a cortex sample of normal or high fat fed mouse. **(E)** For mRNAs with significant expression correlation with lncRNAs in **(C)**, GO analysis was performed. Top 10 most highly enriched terms are shown based on their FDR *q*-values.

Using the same RNA sequencing data analyzed above, we examined expression changes of lncRNAs in brain cortexes from high fat diet fed mice (Supplementary Table S3). Many lncRNAs were changed in their expression (Figure 3A). We measured the expression of six randomly selected lncRNAs by PCR and confirmed the same pattern of expression change with RNA sequencing data (Figure 3B). Among lncRNAs in this list, roles for several lncRNAs have been reported. Genomic inspection of RP23-143A14.3 suggests that this lncRNA is the possible host gene of miR-212∼132. These miRNAs are necessary for proper neural development. Their dysregulation can lead to a neurological disorder (Wanet et al., 2012). Miat was initially reported as the lncRNA involved in myocardial infarction. It is also a lncRNA important for brain development (Ishii et al., 2006; Aprea et al., 2013). However, no previous study has shown any connection of those differentially expressed lncRNAs to brain metabolism (Figure 3A).

We analyzed genomic locations of lncRNAs identified above and found that locations of promoter regions for two lncRNAs, RP24-131B6.1 and RP24-209I7.2, were overlapping with their neighboring protein-coding genes, Degs1 and Sox8, respectively, in the opposite direction in genomic context (Figure 3C). Previous studies about lncRNAs have shown that many lncRNA-mRNA pairs with this kind of relationship have correlated expression pattern and related functions (Guo et al., 2015; Zhang et al., 2018). Thus, we checked expression levels of neighboring mRNAs. We found that there is high expression correlation for those selected lncRNA-mRNA pairs (Figure 3D). Thus, these lncRNA-mRNA pairs might be under the control of the same signaling pathway to be regulated together. Degs1, a dihydroceramide desaturase, is involved in the ceramide biosynthetic pathway which is implicated in neurodegeneration (Jana et al., 2009). Sox8 has a role in terminal differentiation of oligodendrocytes (Stolt et al., 2004). Interestingly, concordant expression between Sox8 and RP24-209I7.2 (also termed as Sox8OT) has been reported previously (Mercer et al., 2010). Although roles of Degs1 and Sox8 have not been reported in high fat diet-induced animal model, they might be involved in brain metabolism under high fat diet fed condition based on their differential expression found in our data.

To surmise possible functions of those selected lncRNAs, we collected mRNA genes with strong correlation in expression with these lncRNAs, respectively (Supplementary Table S4). We performed GO analysis for each mRNA group. Strikingly, myelin sheath was identified as the most enriched GO term for both lncRNAs (Figure 3E). In the central nervous system, oligodendrocytes are related to the formation of myelin sheath to electrically insulate axons and support axon metabolism (Hirrlinger and Nave, 2014). Loss of myelin has critical effects on neurological disease including multiple sclerosis and stroke (Franklin and Goldman, 2015; Armstrong et al., 2016). It also plays a role in obesity contributing to cognitive decline (Kullmann et al., 2015). Recent studies have demonstrated that high fat diet intake could trigger demyelination in the brain (Guerrero-Garcia et al., 2016; Raddatz et al., 2016). Among the differentially expressed genes related with myelination, we confirmed the decrease of a myelination marker Mog (myelin oligodendrocyte glycoprotein) in the brain cortex from the mice fed with high fat diet (Duvanel et al., 2003) (Figure 2B). Taken together, these evidences suggest that high fat diet can trigger demyelination in the cortex and ultimately contribute to memory loss, possibly through the regulation of the pathway involved with the lncRNAs, RP24-131B6.1 and RP24-209I7.2.

### Analysis of circRNAs Change in the Brain Cortex After High Fat Diet

**FIGURE 4:**
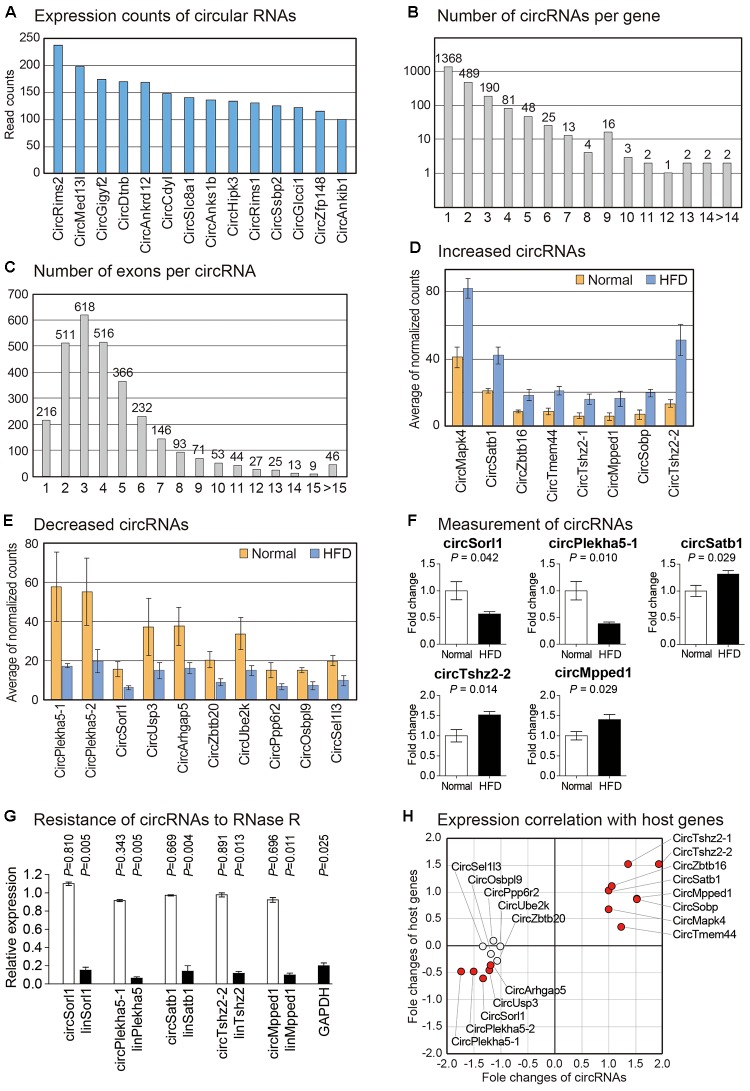
Analysis of circRNAs in the brain cortex of mouse fed with high fat diet. **(A)** Read counts of circRNAs most highly expressed in brain cortex samples are shown. Only those circRNAs with average expression counts higher than 100 are shown. **(B)** Number of circRNAs with different combination of exons per each gene is counted. For most genes, only single type of circRNA is expressed. **(C)** Number of exons comprising each circRNA is counted. Most circRNAs comprise two to four exons. **(D,E)** List of significantly increased **(D)** or decreased **(E)** circRNAs in the brain cortex from high fat fed mouse. For each gene, average values of normalized circRNA counts in each group were calculated. **(F)** Measurement of circRNAs by PCR. Five circRNAs were randomly selected among those in **(D,E)** to confirm their expression change. The data in Supplementary Figure S4A were used for the quantitation. *P*-value was calculated by two-tailed *t*-test (*n* = 4 in each group). **(G)** The circular structure of circRNAs was confirmed by RNase R treatment. The data in Supplementary Figure S4B were used for the quantitation. White bars indicate circRNAs while filled bars mean linear RNAs. *P*-value was calculated by comparing the expression levels between untreated and RNase R-treated samples with two-tailed *t*-test (*n* = 3 in each group). **(H)** For circRNAs in **(D,E)**, expression correlations with their host genes are shown. Fold changes of each circRNA and its host gene were used to depict the scatter plot.

Many studies have shown that circRNAs are important regulators in diverse biological processes, especially in the brain (Rybak-Wolf et al., 2015; You et al., 2015; Shao and Chen, 2016). To analyze expression changes of circRNAs in brain cortexes of high fat fed mice, we used DCC algorithm to detect reads spanning back-splice junction from RNA sequencing data reflecting sequences of circRNAs (Cheng et al., 2016). We calculated expression counts of circRNAs and presented highly expressed circRNAs in the samples we analyzed (Figure 4A and Supplementary Table S5). We then calculated the number of circRNAs with different exon composition generated from each gene. About two-thirds of circRNAs-coding host genes expressed a single form of circRNA (Figure 4B and Supplementary Table S6). Interestingly, diacylglycerol kinase iota (Dgki) produced circRNAs with 23 different forms, suggesting a complex regulation for the production of circRNAs from this gene (Supplementary Table S6). We also analyzed the number of exons used to produce each circRNA. More than half of circRNAs were composed of two to four exons (Figure 4C and Supplementary Table S7), similar to results of previous circRNAs studies (Rybak-Wolf et al., 2015; Xu et al., 2017). The circRNA with the highest number of exons (36 exons) was produced from tetratricopeptide repeat domain 3 (Ttc3) gene (Supplementary Table S7).

Using normalized counts of circRNAs, we analyzed circRNAs with differential expression pattern between brain cortexes of normal and high fat fed mice. We identified eight increased and ten decreased circRNAs, respectively (Figure 4D,E). No study has reported the function of these circRNAs. Host genes of these circRNAs in high fat diet-induced model have no reported study either, although Sorl1, the host gene of circSorl1, has been shown to be genetically associated with AD (Rogaeva et al., 2007). We confirmed the expression change of five randomly selected circRNAs by PCR. All of these circRNAs showed a significant change in their expression (Figure 4F). Moreover, their resistance to RNase R treatment and the confirmation of back-splice junction through Sanger sequencing suggested that those circRNAs indeed exist as a circular structure in the cells (Figure 4G).

Previous studies have reported that there are different degrees of correlation between the expression of circRNA and its host gene depending on samples used (Okholm et al., 2017; Jiang et al., 2018). Therefore, we calculated expression correlation for differentially expressed circRNAs selected above with their host genes (Figure 4D,E). We found that many circRNA-host gene pairs showed high correlations in their expression (Figure 4H). Thus, circRNA might be involved in the common signaling pathway with its host gene in our model, thereby acting as a regulatory molecule.

## Discussion

It is known that high fat diet contributes to cognitive dysfunction by reducing synaptic plasticity, aggravating neuroinflammation, and suppressing LTP in brain cortex (Uranga et al., 2010; Noble et al., 2011; Val-Laillet et al., 2011). Although many studies have shown expression change of protein-coding genes in high fat diet (Val-Laillet et al., 2011; Kothari et al., 2017), the role of non-coding RNAs in the brain cortex from high fat diet fed model has not been reported yet. In the present study, we analyzed expression of both coding and non-coding RNAs in high fat diet fed brain cortex to provide comprehensive transcriptome profiles. We found that protein-coding genes related to neurogenesis, synaptic plasticity, and calcium signaling showed the most significantly altered pattern in high fat diet fed brain cortex. We also analyzed expression profiles of lncRNAs and circRNAs in the same samples. Our analyses showed dramatic differential expression of lncRNAs and circRNAs in high fat diet fed brain cortex.

In our analysis of differentially expressed mRNAs, neurogenesis and synapse were two most highly enriched GO terms in high fat diet fed mouse cortex. We found decreased expression of Synaptotagmins (Syt families) including Syndig1l, Syt6, Syndig1, and Sytl5 (Supplementary Tables S1, S2). Syt families are associated with synaptogenesis and neurotransmitter release (DiAntonio and Schwarz, 1994) and promote the formation of axonal branches in forebrain neurons (Greif et al., 2013). Thus, high fat diet could lead to impairment of synaptic plasticity in part by reducing the expression of synapse-related proteins such as Syt families.

We noticed expression changes of several important neuronal receptors in the brain cortex after high fat diet. The NMDA receptor is critical for glutamatergic neurotransmission and synaptic plasticity by inducing long term depression (LTD) in learning and memory processes such as recognition memory (Cooke and Bliss, 2006; Warburton et al., 2013). NMDA receptors consist of two obligatory GluN1 (NR1) subunits, two subunits of GluN2 (NR2A, NR2B, NR2C, NR2D), and two GluN3 (NR3A and NR3B) subunits that all bind glutamate (Paoletti et al., 2013). The expression of NR2 subunits plays critical roles in synaptic plasticity (Yashiro and Philpot, 2008). It was shown that the deletion of NR2C in mouse resulted in alternation in the synaptic transmission (Ebralidze et al., 1996). We verified the decreased expression of NR2C (encoded by Grin2c) in high fat diet fed brain cortex (Figure 2B). The decrease of NR2C expression in high fat diet fed mouse brain cortex might play a role in the synaptic dysfunction and the reduction of LTP and LTD, leading to cognitive decline in high fat diet brain.

Previous reports have shown the role of lncRNAs and circRNAs in the brain. For example, the role of several lncRNAs have been identified in neuronal development and disease (Mercer et al., 2010; Aprea et al., 2013; Ng et al., 2013). One of the initially identified circRNA, Cdr1as, has been shown to be essential for maintaining normal brain function (Hansen et al., 2013; Piwecka et al., 2017). Although circRNAs are recognized as important regulators, no study has profiled circRNAs affected by high fat diet. No study has reported the expression of lncRNAs in the brain from high fat diet fed mouse either. Our study is the first one that comprehensively profiles two important regulatory non-coding RNAs, lncRNAs and circRNAs, in high fat diet-induced animal model.

Myelin sheath is generated from oligodendrocytes and critical for saltatory conduction between neurons (Young et al., 2013). Active myelination remodeling is necessary for learning and memory processes (McKenzie et al., 2014). From expression analysis of genes related to lncRNAs, RP24-131B6 and RP24-209I7.2, we also observed decreased expression of diverse myelination-related genes in addition to Mog in high fat diet fed mouse brain cortex (Figure 2B). This includes Plp1 (proteolipid protein 1), Gsn (gelsolin), Cnp (2′, 3′-cyclic nucleotide 3′ phosphodiesterase), Pllp (plasmolipin), and Mobp (myelin-associated oligodendrocyte basic protein) (Supplementary Tables S3, S4). Plp1 is a transmembrane protein holding myelin. It is associated with abnormal axonopathy (Inoue, 2005). Pllp (Hasse et al., 2002) and Mobp (Gould et al., 1999) in oligodendrocytes are proteins for formatting myelination in the brain (Mitkus et al., 2008). Because the expression of these genes has a high correlation with lncRNAs, RP24-131B6 and RP24-209I7.2, they might be under the control of a common signaling pathway. Elucidating this signaling network is a future direction of this study.

## Conclusion

Our study presents comprehensive transcriptome profiles of high fat diet fed mouse brain cortex. Our study is the first study that analyzes both coding and non-coding RNAs in this model. The profile of non-coding RNAs in high fat diet fed brain will be helpful for future studies about cognitive dysfunction resulted from high fat diet-induced dementia. Thus, this study provides important information to understand roles of diverse RNAs in high fat diet-induced neuropathology.

## Data Availability

The sequencing data is available through the Gene Expression Omnibus database.

## Author Contributions

GY, KC, and JS performed the biochemical and animal experiments. Y-KK performed the bioinformatics analysis. JS and Y-KK wrote the manuscript.

## Conflict of Interest Statement

The authors declare that the research was conducted in the absence of any commercial or financial relationships that could be construed as a potential conflict of interest.

## Footnotes

1 http://www.bioinformatics.babraham.ac.uk/projects/fastqc/

## Supplementary Material

FIGURE S1 Increase of weight and impairment of glucose homeostasis for the mice with high fat diet. **(A)** The weight of ten mice with normal diet and that of ten mice with high fat diet was compared. We randomly selected four mice for the transcriptome analysis from each group. **(B)** The glucose tolerance test was performed. For the test, five mice were used in each group. *P*-value was calculated by two-tailed *t*-test at each time point.

FIGURE S2 Western blot data used for the quantitation in Figure 1B, 2B. The brain cortexes from two mice fed with normal diet and those from two mice fed with high fat diet were used to extract proteins.

FIGURE S3 Gel images used for the quantitation in Figure 3B. The brain cortexes from four mice fed with normal diet and those from four mice fed with high fat diet were used to measure the expression of lncRNAs.

FIGURE S4 Gel images used for the quantitation in Figure 4F,G. **(A)** The same samples used for the measurement of lncRNAs were used to measure the expression of circRNAs. **(B)** The level of circRNAs and linear RNAs was compared between the untreated (-) and the RNase R-treated (R) samples. Representative images were shown from triplicate experiments. Expected size of PCR band is indicated.

TABLE S1 List of protein-coding genes with their average expression level and fold change between the cortex from normal and high fat diet fed mice. Only those mRNAs with expression level higher than thresholds in both STAR-cuffnorm and Salmon-edgeR pipelines were selected. In STAR-cuffnorm, the mRNAs with average Fragments Per Kilobase of transcript per Million mapped reads (FPKM) value higher than 1 and without FPKM value of 0 in any samples were selected. In Salmon-edgeR, the mRNAs with log value of Counts Per Million (CPM) higher than 0 were selected. For the selection of mRNAs with significant expression change, the mRNAs changed more than 50% in its expression level and *P*-value less than 0.005 were selected in STAR-cuffnorm pipeline. For Salmon-edgeR pipeline, the mRNAs changed more than 50% in its expression level and top 5% mRNAs based on its *P*-value were selected. The mRNAs selected in both pipelines were combined for the following analysis.

TABLE S2 Among the genes with significant expression change in high fat diet group compared to normal group, as selected in Supplementary Table S1, those related to neurogenesis, synapse, and calcium signaling were shown with their fold changes and *P*-values.

TABLE S3 List of lncRNAs with their average expression level and fold change between the cortex from normal and high fat diet fed mice. The lncRNAs with expression level higher than thresholds in both pipelines were selected with the same criteria applied for mRNAs as in Supplementary Table S1. For the selection of lncRNAs with significant expression change, the lncRNAs changed more than 40% in its expression level and *P*-value less than 0.1 were selected in STAR-cuffnorm pipeline. For Salmon-edgeR pipeline, the lncRNAs changed more than 40% in its expression level and top 10% lncRNAs based on its *P*-value were selected. The lncRNAs selected in both pipelines were combined for the following analysis.

TABLE S4 List of genes used for the analysis in Figure 3E. The protein-coding genes with expression correlation higher than 0.95 with each lncRNA (highlighted with green color) were used for gene ontology (GO) analysis, respectively.

TABLE S5 List of circRNAs with their genomic locus, information of host gene, and expression counts in each sample are shown. Only the circRNAs with average counts higher than 2 were selected. **(B)** List of circRNAs with normalized counts and fold change between the cortex from normal and high fat diet fed mice are shown. Among the circRNAs in **(A)**, only those with normalized counts higher than 10 were selected.

TABLE S6 For each host gene of circRNAs, the number of detected circRNAs with different exon composition is counted. This data is used to depict the graph in Figure 4B.

TABLE S7 The number of exons used to produce each circRNA is counted. This data is used to depict the graph in Figure 4C.

TABLE S8 Information of antibodies for western blot and primer sequences for PCR.
