# Supplementary figures and images for: Transcriptomic Analysis of High Fat Diet Fed Mouse Brain Cortex
Source: Front Genet. 2019 Feb 19;10:83. doi: 10.3389/fgene.2019.00083 (PMC6389608; doi:10.3389/fgene.2019.00083)

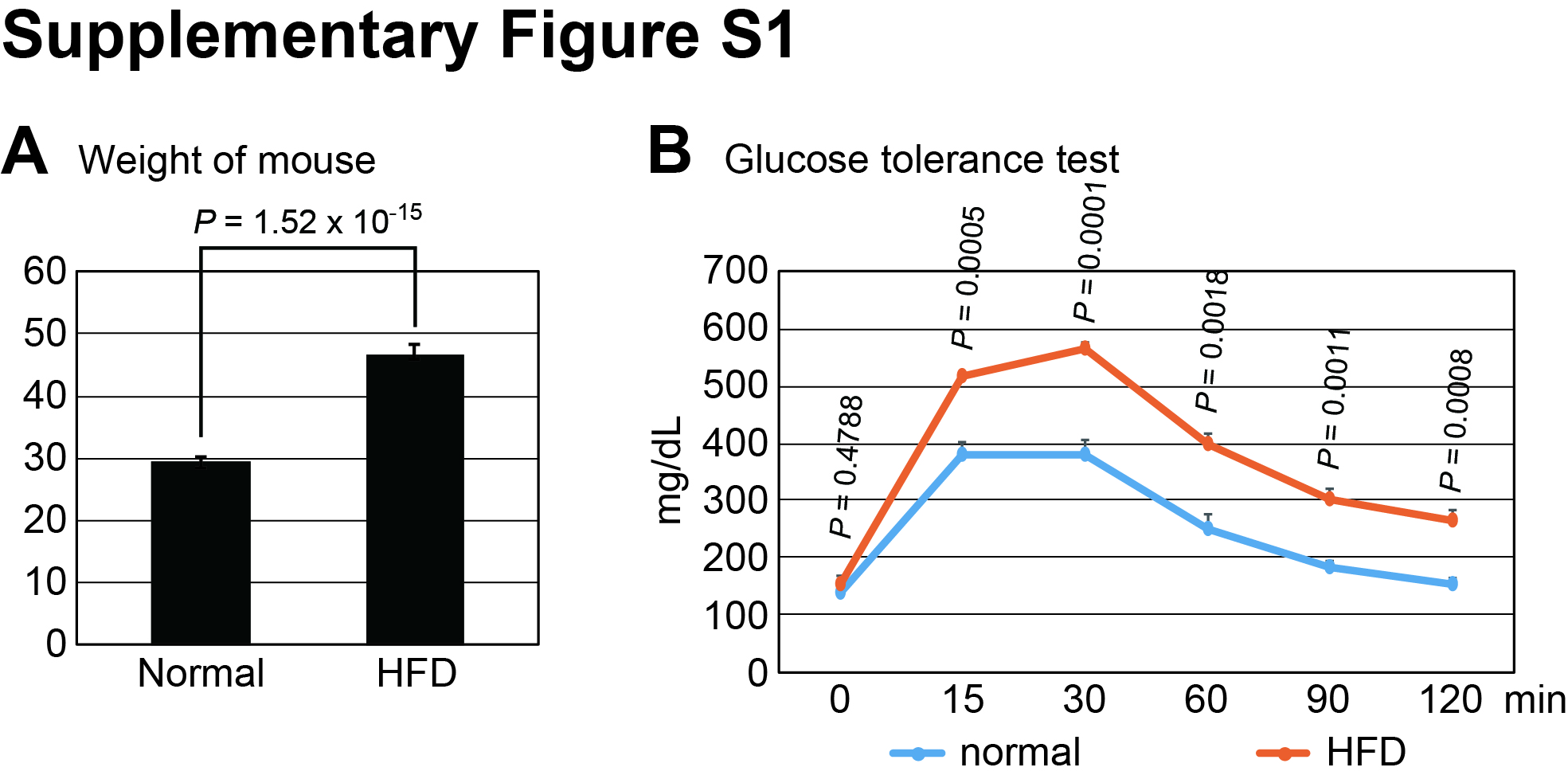

Supplement: FIGURE S1 — Increase of weight and impairment of glucose homeostasis for the mice with high fat diet. (A) The weight of ten mice with normal diet and that of ten mice with high fat diet was compared. We randomly selected four mice for the transcriptome analysis from each group. (B) The glucose tolerance test was performed. For the test, five mice were used in each group. P-value was calculated by two-tailed t-test at each time point. [file Image_1.JPEG]

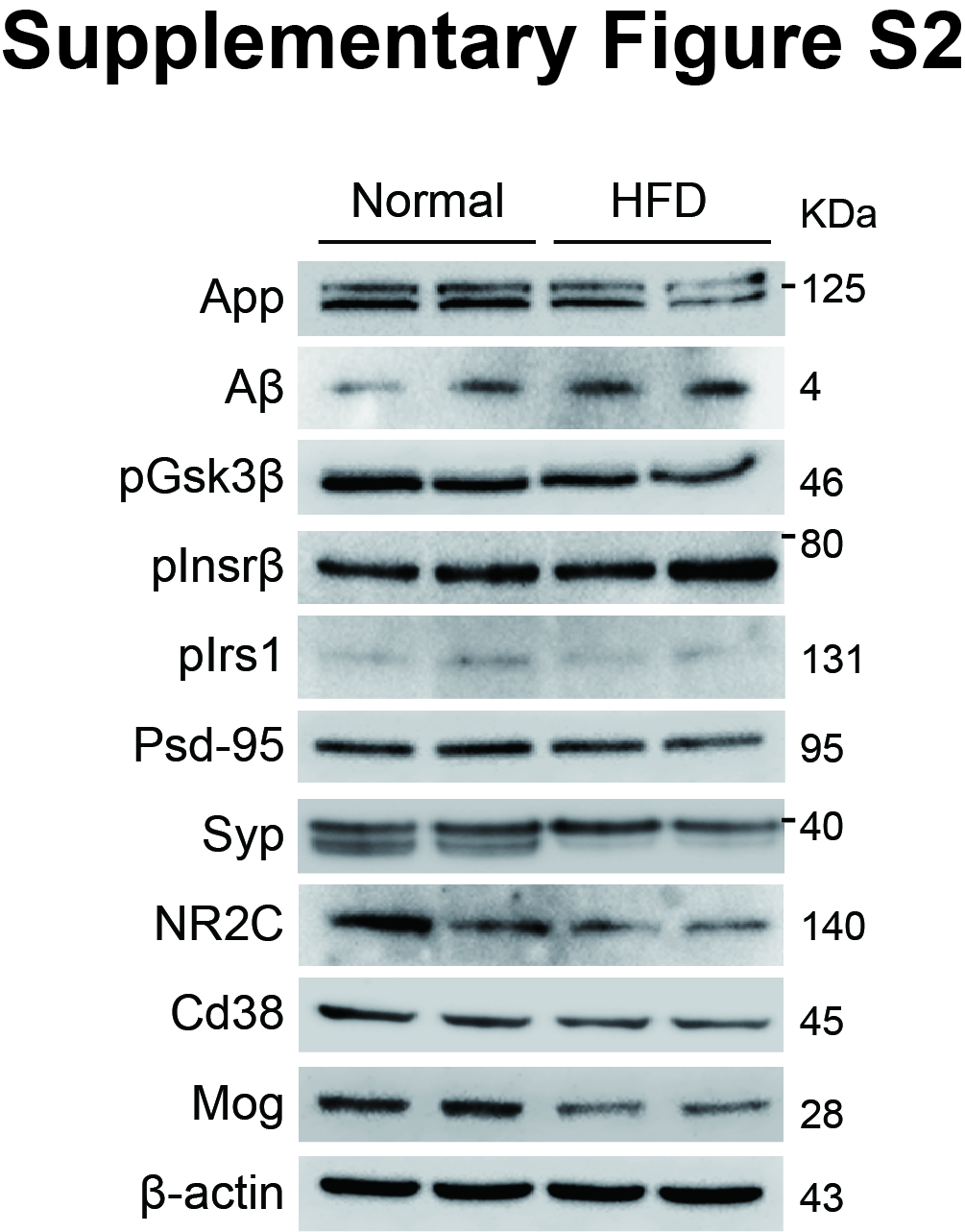

Supplement: FIGURE S2 — Western blot data used for the quantitation in Figure 1B, 2B. The brain cortexes from two mice fed with normal diet and those from two mice fed with high fat diet were used to extract proteins. [file Image_2.JPEG]

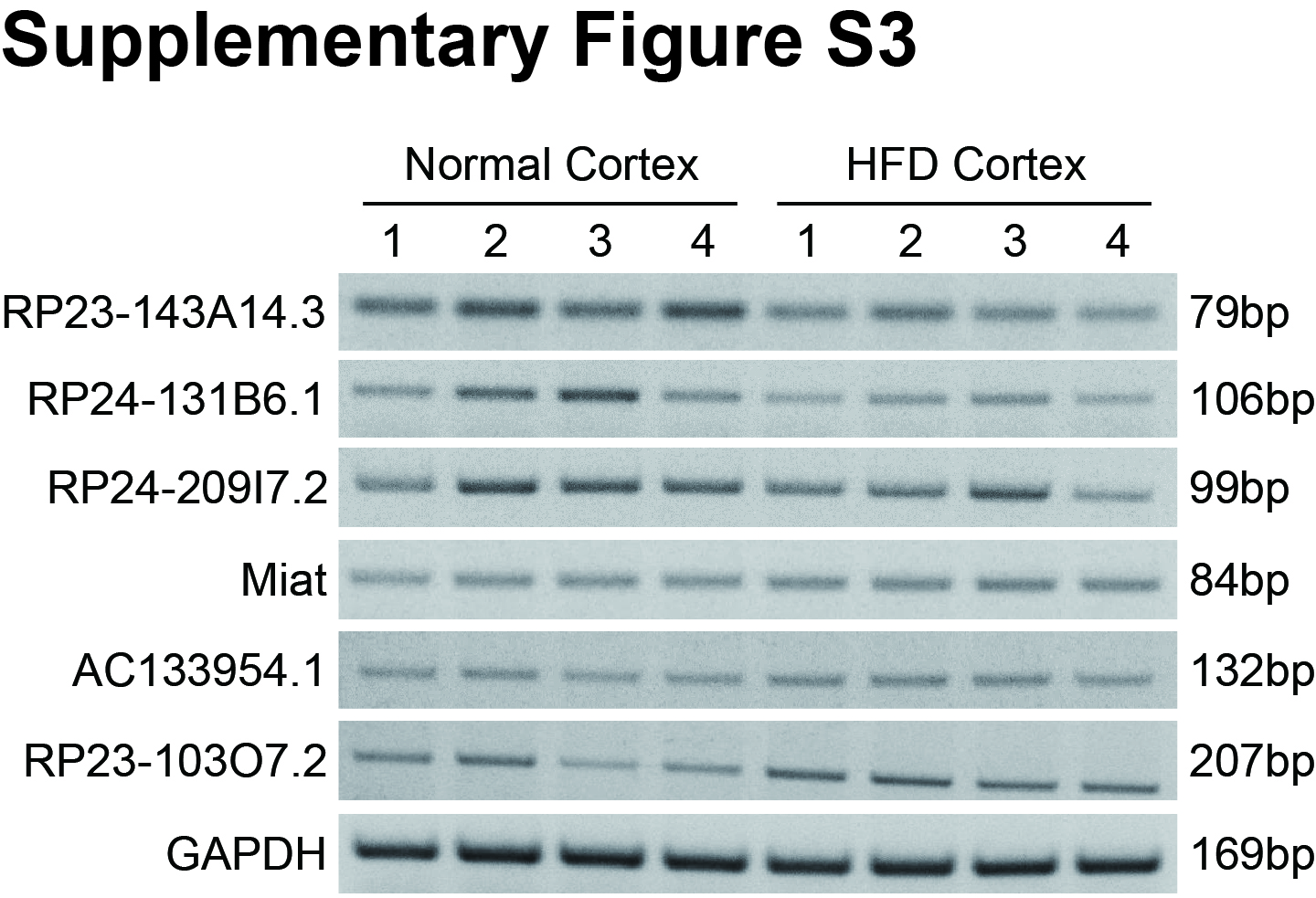

Supplement: FIGURE S3 — Gel images used for the quantitation in Figure 3B. The brain cortexes from four mice fed with normal diet and those from four mice fed with high fat diet were used to measure the expression of lncRNAs. [file Image_3.JPEG]

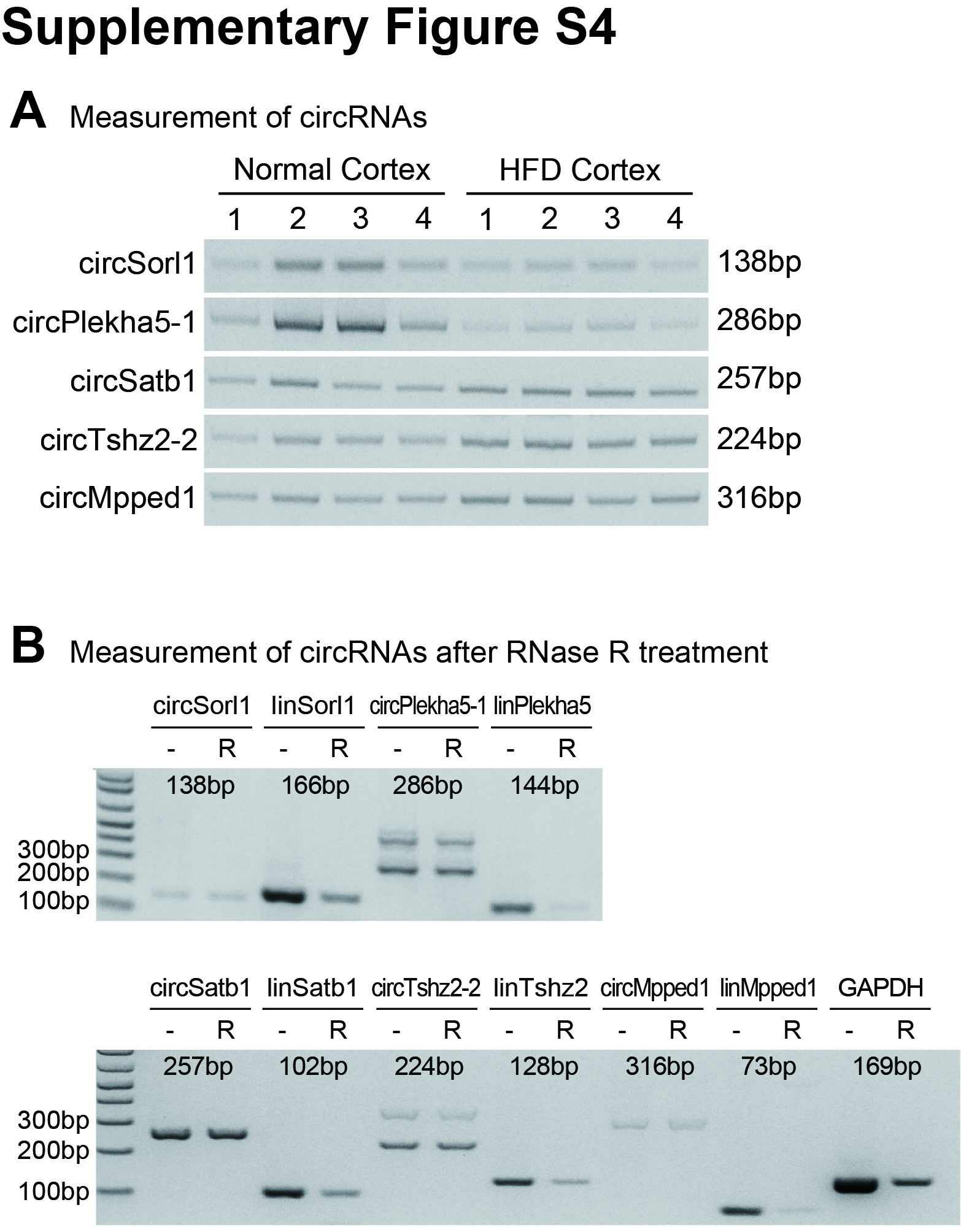

Supplement: FIGURE S4 — Gel images used for the quantitation in Figure 4F,G. (A) The same samples used for the measurement of lncRNAs were used to measure the expression of circRNAs. (B) The level of circRNAs and linear RNAs was compared between the untreated (-) and the RNase R-treated (R) samples. Representative images were shown from triplicate experiments. Expected size of PCR band is indicated. [file Image_4.JPEG]
